# Preseason Body Composition Phenotypes and In-Season Injury Burden in Male Professional Basketball: A Retrospective Cohort Study

**DOI:** 10.3390/sports14030122

**Published:** 2026-03-20

**Authors:** Javier Pérez-Murillo, Pedro Cotolí-Suarez, Borja Ricart-Luna, Vicente Sebastià Alcacer, Álvaro Domínguez García, Marcelino Pérez-Bermejo, María Teresa Murillo-Llorente, Eloy Jaenada-Carrilero

**Affiliations:** 1 SONEV Research Group, Faculty of Medicine and Health Sciences, Catholic University of Valencia San Vicente Mártir, C/Quevedo No. 2, 46001 Valencia, Spain; marcelino.perez@ucv.es (M.P.-B.); mt.murillo@ucv.es (M.T.M.-L.); 2 Alqueria Lab Department, Valencia Basket, 46013 Valencia, Spain; pcotoli@alqueriadelbasket.com (P.C.-S.); bricart@alqueriadelbasket.com (B.R.-L.); vicen1@msn.com (V.S.A.); adominguez@valenciabasket.com (Á.D.G.); 3 Department of Physiotherapy, Faculty of Medicine and Health Sciences, Catholic University of Valencia San Vicente Mártir, C/Ramiro de Maeztu, 19, 46900 Valencia, Spain; eloy.jaenada@ucv.es

**Keywords:** athletic injuries, body composition, anthropometry, risk factors, sports medicine, exercise-related injuries

## Abstract

Professional basketball entails high physical demands and a complex injury profile in which injury burden and time-loss distribution critically affect player availability. This study explored the association between preseason anthropometric body composition and in-season injury burden in male professional basketball and explored body phenotypes linked to greater injury accumulation. A retrospective longitudinal cohort design was applied using official injury records and standardized ISAK anthropometric assessments collected during preseason. Players from two male professional teams (first team, ACB; second team, LEB Plata) were included. Outcomes were the number of injuries and observed days lost during the season. Associations were assessed using Pearson correlations, principal component analysis (PCA), team-stratified logistic regression, and unsupervised k-means clustering. Injury burden demonstrated a highly skewed distribution, with a small subset of players accounting for a large proportion of total days lost. Preseason adiposity markers showed strong internal coherence, with PCA identifying a dominant component reflecting an adiposity gradient. Higher preseason body fat percentage was associated with a greater likelihood of high injury burden (≥3 injuries/season) in both teams. Clustering revealed two phenotypes: a higher-adiposity, higher-burden profile and a lower-adiposity, lower-burden profile. These exploratory findings suggest that preseason body composition, particularly adiposity, may be related to injury burden in male professional basketball. However, given the limited sample size and exploratory design, the results should be interpreted cautiously and considered hypothesis-generating. Precompetitive body phenotyping may therefore provide preliminary information for identifying players potentially at elevated risk of recurrent injury accumulation and reduced competitive availability.

## 1. Introduction

Professional basketball is one of the team sports with the highest competitive density and a high neuromuscular demand, which translates into a multifactorial injury profile characterized by a combination of acute trauma and overuse injuries, predominantly muscular and ligamentous, with special involvement of the lower extremity. Epidemiological studies in professional teams have shown high incidences in both training and, especially, competition, with a predominance of muscular and ligamentous injuries of the lower extremity, particularly at the ankle, knee, and hamstring and adductor musculature [1,2,3,4]. Recent analyses in teams from Liga Endesa and other national competitions have confirmed that accumulated exposure, calendar congestion, and match density constitute relevant determinants of injury burden throughout the season [1,2]. In parallel, the evolution of modern basketball toward a higher-intensity game, with faster possessions and a high frequency of explosive actions (jumps, accelerations, changes in direction, and contacts), increases locomotor demands and could amplify injury risk [3,5]. Injury in professional basketball should be interpreted fundamentally as a problem of competitive availability, since its impact is not distributed homogeneously among players: a substantial proportion of days lost may be concentrated in a small number of athletes, disproportionately affecting overall team availability across the season.

Body composition is recognized as a fundamental determinant of performance in professional basketball. Professional basketball players are characterized by tall stature and high body mass, with relatively low fat mass values, but with marked variability according to playing position. In a large study of players from Spanish leagues, Vaquera et al. observed that athletes from the Association of Basketball Clubs (Spanish acronym: ACB) presented higher body fat percentages than those from lower categories (13.0% vs. 10.5%), together with relevant differences among guards, forwards, and centers in body circumferences and skinfolds [6]. Other studies have confirmed that centers present greater body mass, height, and body fat percentage, whereas perimeter players tend to show lower adiposity and different profiles of power and aerobic capacity [7,8,9].

Players’ body composition is also dynamic throughout the season. In a longitudinal study of high-level junior players, significant fluctuations in fat mass and lean mass were observed according to the competitive period, with notable changes between preseason and phases of high load [10]. In this sense, preseason constitutes a strategic window to establish a basal profile of health and performance and, potentially, to identify profiles with greater vulnerability before the accumulation of fatigue and competitive exposure.

Available evidence in other sporting contexts supports the hypothesis that adiposity may act as an injury risk factor. A recent systematic review in young athletic populations concluded that a high BMI is associated with a greater risk of injury, especially in the lower extremity, with an overall OR of 1.18 for non-bone injuries [11]. It has also been reported that a higher percentage of fat mass is related to a history of knee injury in adolescent athletes [12]. Similarly, in military studies, recruits with poorer physical fitness or who exceeded body fat thresholds presented more musculoskeletal injuries and a higher risk of stress fractures [13]. However, extrapolation to professional basketball requires caution, since the relationship between adiposity and injury may manifest not only as increased incidence, but also as a greater probability of accumulating repeated episodes or a higher temporal burden in certain player subgroups, which would have direct implications for competitive availability.

In professional team sports, models incorporating body composition as an injury predictor are beginning to emerge. In soccer, certain anthropometric and physical fitness variables have been observed to predict the probability of injury during the season [14]. In basketball, Bertuccioli et al. [15] showed that segmental bioelectrical parameters (BIVA) and body composition are associated with injury incidence and severity during training and competition. However, evidence remains scarce, with small samples, methodological heterogeneity, and limited standardization in anthropometric assessment. Moreover, much of the literature focuses on injury occurrence as a binary event or on total injury counts, whereas in the professional context, it is particularly relevant to identify the subgroup with high injury burden (for example, repeated accumulation of injuries during the season), due to its impact on collective performance and medical-nutritional planning.

Despite the relevance of the problem, there are no published studies that systematically analyze the relationship between preseason anthropometric body composition and injury incidence across a complete season in a professional basketball team using standardized anthropometry. Therefore, this study aims to explore the potential association between preseason body composition (measured by standardized anthropometry) and injury burden recorded throughout a competitive season in professional basketball players, with the broader objective of identifying possible body composition patterns associated with greater injury accumulation that may inform preventive, nutritional, and load-management strategies. Given the exploratory nature of the analytical approach, including dimensionality reduction and clustering techniques, the study is guided by the working hypothesis that players with a body composition phenotype characterized by greater total and central adiposity during the preseason period may present a higher probability of accumulating a higher injury burden during the season compared with players with lower adiposity profiles.

## 2. Materials and Methods

### 2.1. Study Design

An analytical observational study with a retrospective longitudinal design was conducted, based on secondary analysis of data previously recorded at Valencia Basket Club. The investigation was supported by two main sources: (i) official clinical injury records collected by the club’s medical and physiotherapy services during the competitive period and (ii) anthropometric measurements performed in preseason using a standardized anthropometry protocol of the International Society for the Advancement of Kinanthropometry (ISAK) [16] by accredited personnel. This was a non-interventional study, in which no modifications were introduced in training load, sport planning, or routine clinical practice.

### 2.2. Study Population and Sample

During the analyzed season, the target population consisted of professional players belonging to two male rosters of Valencia Basket Club: the first and second men’s teams. Players officially registered on either roster, with complete preseason anthropometric assessment available and with injury and time-loss records during follow-up, were included.

Players without complete preseason anthropometry, with incomplete or non-interpretable injury or time-loss records, as well as those who did not complete the season with the club due to contractual or sporting reasons (e.g., loans, releases, or temporary signings) that prevented homogeneous follow-up, were excluded.

Regarding sample size, the initial estimation was based on the usual roster size in both categories, with an approximate range of 12–15 players in the first team and 12–14 players in the second team. Given the retrospective nature of the study, the final sample size was conditioned by the availability and completeness of anthropometric and injury records. All players who met the inclusion criteria had complete preseason anthropometric assessments and injury follow-up records; therefore, no additional exclusions were required.

Due to the inherent constraints of single-team professional cohorts, the resulting sample size is necessarily limited and does not allow strong statistical inference. At the methodological level, the study was designed to provide a complete descriptive characterization of the cohort and to explore potential associations through correlations and simple statistical models. Due to the inherent constraints of studies conducted within professional team environments, the available sample size is necessarily limited by roster composition. In the present study, the cohort consisted of players from two male professional rosters within the same club (first team and second team). Consequently, the final sample size reflects the complete population of eligible players available during the analyzed season. This limitation should be considered when interpreting the statistical analyses performed in the study.

### 2.3. Study Variables

#### 2.3.1. Independent Variables: Preseason Body Composition

Independent variables corresponded to anthropometric parameters collected in preseason according to the ISAK protocol. Body mass (kg) and height (cm), as well as body mass index (BMI, kg/m^2^), were included. Skinfolds (mm) were recorded following the standardized protocol, including at least triceps, biceps, subscapular, iliac crest, supraspinale, abdominal, anterior thigh, and calf, with calculation of the sum of skinfolds (S8, mm). Likewise, body circumferences (cm) such as relaxed arm, contracted arm, thigh, and calf were included.

From these measurements, derived variables aimed at characterizing body composition and regional adiposity distribution were calculated. These included body fat percentage estimated using the Faulkner anthropometric equation based on the sum of four skinfolds (triceps, subscapular, supraspinale, and abdominal), which is commonly applied in sports settings within the ISAK framework. Fat-free mass (kg) and other derived body indices were subsequently calculated according to standard anthropometric procedures [17,18].

#### 2.3.2. Dependent Variables: Sports Injuries and Injury Burden

Dependent variables were extracted from the club’s official clinical registry. The primary outcome was injury burden during the season, operationalized through the total number of injuries per player and the total sum of days lost. Days lost were counted from the date of injury diagnosis to the officially recorded return-to-performance date (sport clearance) by the club’s medical services.

Additionally, when available, each injury was classified by clinical type (muscular, ligamentous, bone, tendinous, or joint) and by anatomical location (ankle, knee, thigh, leg, foot, hip, lumbar region, shoulder, and hand) following procedures agreed upon in the sports injury surveillance literature [19,20]. Likewise, severity categorization according to time loss was considered, with the usual stratification into mild (<7 days), moderate (8–28 days), and severe (>28 days) injuries, following criteria widely employed in sports epidemiological studies [21] and international recommendations for injury recording in sport [22].

As a secondary dependent variable for predictive analysis, the outcome “high injury burden” was defined, operationalized as the occurrence of three or more injuries during the season (≥3 injuries). This threshold was selected to identify players presenting a recurrent accumulation of injury episodes within a single competitive season, distinguishing them from players with isolated or occasional injuries. In the present cohort, this cut-off corresponded to the upper segment of the observed injury distribution, allowing the identification of players with a disproportionate contribution to total injury burden and time-loss.

Therefore, the ≥3 injuries threshold should be interpreted as an analytical criterion aimed at distinguishing recurrent injury accumulation rather than as a universal clinical definition of high injury burden. This approach is based on studies addressing the occurrence and classification of recurrent injuries in sports research [23,24], and on methodological recommendations emphasizing the importance of considering accumulated injury burden as a clinically meaningful epidemiological outcome, beyond simple incidence [25].

### 2.4. Data Sources and Collection Procedures

#### 2.4.1. Anthropometric Procedure

Anthropometric measurements were performed in preseason, during the first weeks of the preparatory cycle, following a standardized ISAK protocol by an accredited anthropometrist. Calibrated instruments were used, including a skinfold caliper with submillimeter precision, an electronic scale for body mass measurement, and a stadiometer for height, together with an anthropometric tape for circumferences. For skinfolds, each anatomical site was measured in triplicate, and the median was used as the final value for analysis, in order to minimize intra-observer error and increase recording reliability.

#### 2.4.2. Injury Registry

Injury data were extracted from the club’s official clinical registry. Diagnosis was established by healthcare professionals from the Valencia Basket Club medical staff, including physicians specialized in sports medicine and/or clinical physiotherapists responsible for clinical follow-up. For each injury episode, the injury date, the sport clearance date when available, and therefore the corresponding time-loss period were recorded. In cases without a recorded clearance date, the injury was included for episode counting, and the calculation of days lost was handled using a conservative analytical criterion according to data availability.

#### 2.4.3. Confidentiality and Data Management

Data were treated in an anonymized manner through the assignment of numerical codes to each player, ensuring the impossibility of individual identification in the final analytical dataset. No personally identifiable data were shared with third parties. The final dataset was stored on institutional media with access restricted exclusively to authorized investigators, ensuring compliance with data protection regulations and applicable confidentiality guidelines.

### 2.5. Statistical Analysis

Statistical analysis was performed using preseason anthropometric records (August–September) and the official clinical injury registry from the competitive period. Continuous variables were described using mean and standard deviation, and median and interquartile range when distributions were asymmetric. Categorical variables were summarized using frequencies and percentages.

Injury burden was evaluated using two main player-level outcomes: (i) the number of injuries recorded during the season and (ii) observed days lost during the season. Observed days lost were calculated only from injuries with documented sport clearance dates, in order to minimize bias derived from incomplete information in a retrospective design. The distribution of observed days lost was represented using a box-and-whisker plot.

The relationship between preseason body composition and injury burden was explored using a matrix of bivariate correlations (Pearson coefficient), including body fat percentage, sum of skinfolds and representative individual skinfolds, as well as in-season burden outcomes. Coefficients were reported together with their statistical significance values. Prior to correlation analyses, the distribution of variables was visually inspected, and no substantial deviations from linearity were observed; therefore, Pearson correlations were considered appropriate for exploratory assessment of associations.

Prior to multivariate analyses, anthropometric variables were standardized (z-scores) in order to account for differences in measurement scales and to ensure comparability across variables in PCA and clustering procedures.

To characterize body phenotype and reduce dimensionality, principal component analysis (PCA) was applied to preseason body composition variables including body fat percentage, sum of skinfolds (S8), and representative individual skinfolds (triceps, subscapular, supraspinale, abdominal, and thigh), which capture both global adiposity and regional fat distribution. The distribution of players was represented on the plane of the first two components.

As the main predictive analysis, logistic regression models were estimated to evaluate the association between preseason adiposity and high injury burden, defined as ≥3 injuries during the season. Models were adjusted in a team-stratified manner, using preseason body fat percentage as a continuous predictor. Results were expressed as odds ratios (OR) with 95% confidence intervals and *p* values.

Finally, an unsupervised k-means clustering analysis was performed integrating preseason body composition and in-season injury burden measures, with the aim of identifying differentiated risk phenotypes. The number of clusters (k = 2) was selected based on inspection of cluster compactness using the elbow method and on the interpretability of the resulting phenotypes within the context of body composition and injury burden. The resulting groups were graphically represented and their characteristics described using summary statistics.

A *p*-value < 0.05 was considered statistically significant.

Given the relatively limited sample size and the number of outcome events, regression and multivariate analyses were primarily applied as exploratory tools aimed at identifying potential patterns within the dataset rather than establishing definitive predictive models. Accordingly, the results derived from these analyses should be interpreted as exploratory and hypothesis-generating.

### 2.6. Ethical Aspects

The study was conducted in accordance with the principles of the Declaration of Helsinki and was approved by the Research Ethics Committee of the Universidad Católica de Valencia (CEI-UCV) on 16 December 2025, with code UCV/2025-2026/072. Informed consent was obtained from all subjects involved in the study.

## 3. Results

### 3.1. Baseline Characteristics of the Preseason Cohort

A cohort of male professional basketball players with preseason anthropometric assessment (August-September) and injury registry during the competitive season (October-June) was included. The sample comprised 28 players from two professional male teams (first team, *n* = 15; second team, *n* = 13) with anthropometric follow-up and injury records from 08/01/2024 to 06/30/2025. The overall mean age of the cohort was 24.61 years. Players from the first team were older (26.79 ± 4.34 years; range: 18–34) compared with those from the second team (21.22 ± 3.87 years; range: 17–28). Preseason body composition showed considerable interindividual variability, particularly in markers of central adiposity. When comparing both teams, players from the second team presented higher mean values in several adiposity indicators, including body fat percentage and the sum of skinfolds, compared with first-team players (Table 1). These differences suggest the presence of differentiated body composition profiles from the beginning of the competitive cycle.

### 3.2. Injury Epidemiology and In-Season Burden

During the season, injury burden showed a markedly asymmetric pattern. A substantial proportion of players accumulated few or no episodes, whereas a small subgroup accounted for a high number of injuries and, even more importantly, most of the observed days lost. This finding points to a critical phenomenon in professional sport: the competitive impact of injuries is not distributed homogeneously, but rather tends to aggregate in specific profiles (Table 2).

**Figure 1:**
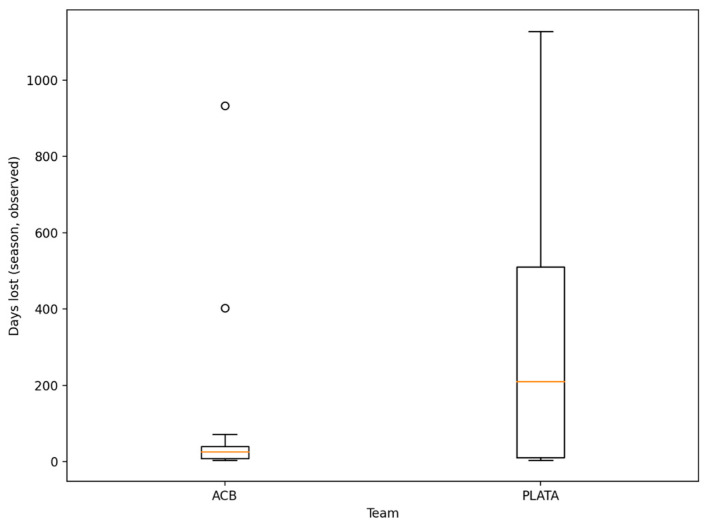
Distribution of observed season days lost (time-loss injuries with documented return date).

The distribution of observed days lost reinforced this concentration pattern, showing a long temporal tail compatible with prolonged absences in a small number of athletes (Figure 1).

### 3.3. Preseason Body Composition Behaves as an Integrated Adiposity Phenotype

Preseason adiposity indicators showed a highly coherent internal structure. Body fat percentage was strongly associated with central skinfolds (subscapular and abdominal) and with other skinfolds, configuring a physiological phenotype of global and central adiposity (Table 3). This pattern supports the internal validity of the anthropometric record and reinforces that the precompetitive body status can be interpreted as an integrated construct rather than as independent measurements.

In contrast, correlations between preseason body composition variables and injury burden outcomes were generally weaker and, in most cases, did not reach statistical significance, suggesting that any potential relationship between adiposity and injury accumulation should be interpreted cautiously in this exploratory cohort.

**Figure 2:**
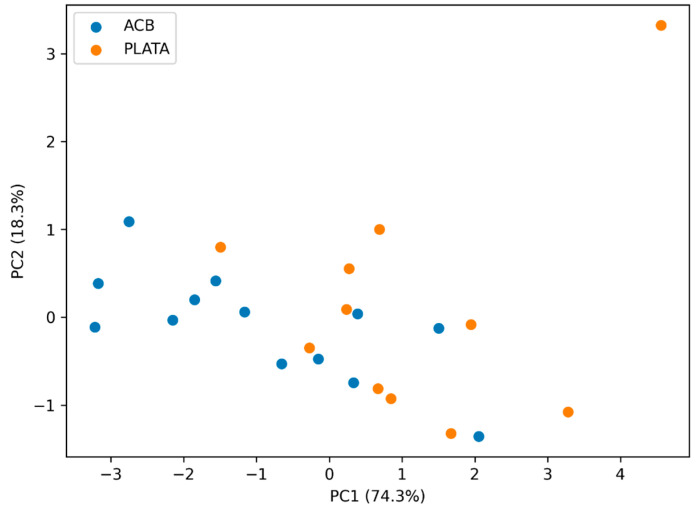
PCA of preseason body composition (PC1-PC2) in the combined cohort.

Principal component analysis confirmed that preseason body composition can be condensed into a dominant axis capturing most of the variability and reflecting a robust adiposity gradient. This result supports the use of a latent body phenotype as a tool to interpret physiological differences in preseason without redundancies due to collinearity among skinfolds (Figure 2).

The most clinically relevant association was observed when redefining the outcome in terms of high injury burden, operationalized as three or more injuries during the season. Preseason body fat percentage showed a positive association with the probability of belonging to the high-burden group, with wide confidence intervals reflecting the limited sample size, suggesting a possible increase in the likelihood of repeated injury accumulation with increasing precompetitive adiposity. However, these estimates should be interpreted cautiously, given the exploratory nature of the analysis and the limited number of cases.

This finding suggests a potential link between a measurable preseason body marker and the injury pattern of greatest competitive impact, that is, the repetition of injury episodes throughout the season.

Given that the competitive context may modify the body composition–injury relationship, team-stratified logistic regression models were estimated. Likewise, because the total injury count captures both isolated and repeated events, a clinically more specific outcome (≥3 injuries) focused on recurrent accumulation was evaluated (Table 4).

The resulting odds ratios should be interpreted cautiously, as the relatively small number of players and outcome events may lead to unstable estimates and wide confidence intervals. Therefore, these results should be considered exploratory indications of a possible association rather than precise effect estimates.

These results suggest that preseason adiposity may be related not only to the occurrence of injuries but also to the accumulation of repeated injury episodes, a scenario that can substantially affect team availability during the competitive season.

### 3.4. Exploratory Clustering: Body Composition Profiles with Divergent Injury Trajectories

**Figure 3:**
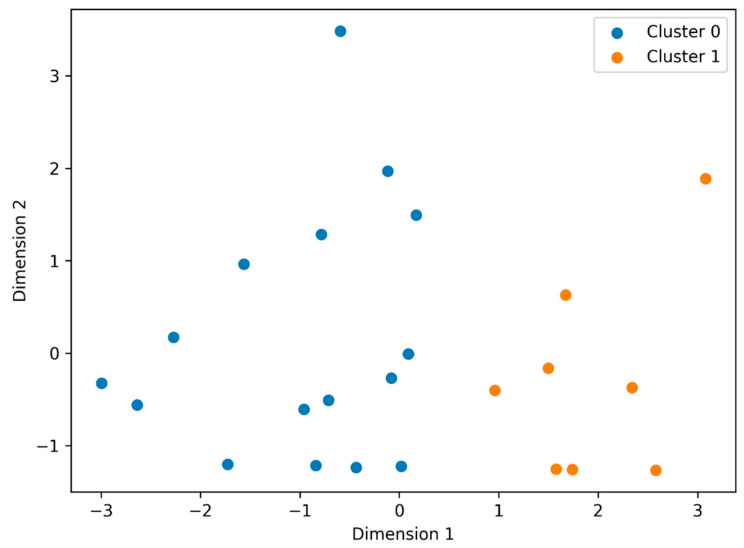
Player clustering based on preseason body composition and season injury burden (k-means, k = 2).

The clustering analysis integrating preseason body composition (global and central adiposity) with in-season injury burden (number of injuries and observed days lost) identified two differentiated exploratory player profiles (Figure 3, Table 5). The higher-burden profile was characterized by higher values of precompetitive adiposity and a more heavily affected competitive pattern, whereas the lower-burden profile grouped more favorable body profiles with lower accumulation of events.

This exploratory grouping emerged from an unsupervised analytical approach, in which player profiles were organized without imposing predefined thresholds or linear relationships. However, given the limited sample size, the stability and reproducibility of the identified clusters should be interpreted cautiously, and the observed player grouping should be considered a preliminary pattern rather than a definitive classification.

Overall, the results show that preseason body composition showed a coherent pattern of global and central adiposity, quantifiable through strong internal correlations and dimensionality reduction by PCA. Injury burden was distributed in a highly asymmetric manner, with concentration in a small subgroup, and clustering analysis identified exploratory profiles with divergent competitive trajectories. Finally, the high-burden outcome (≥3 injuries during the season) showed a positive relationship with preseason adiposity, suggesting that a simple and accessible marker at the beginning of the season may contribute to the identification of players with greater vulnerability to repeated injury accumulation.

## 4. Discussion

This study is a field-based investigation in high-performance sport using real-world data from male professional basketball, showing that body composition assessed in preseason is related to the pattern that most conditions competitive availability: the repeated accumulation of injuries throughout the season. Overall, the results indicate that injury burden is not distributed uniformly, but rather tends to concentrate in a small subgroup of players, and that this vulnerability may be linked to an anthropometric phenotype of global and central adiposity identifiable from the beginning of the competitive cycle.

### 4.1. Injury in Professional Basketball as a Problem of Competitive Availability, Beyond Incidence

The epidemiology of professional basketball consistently describes a complex injury profile and a high incidence of injuries, especially in competition, with a predominance of muscular and ligamentous injuries in the lower extremity [1,2,3,4]. However, in high-performance sport, the operational impact of injuries does not depend solely on the number of episodes, but on their translation into loss of availability (days lost). In our cohort, the distribution of observed days lost evidenced a “long-tail” pattern, compatible with the typical behavior of team sports, in which a small number of players concentrates a substantial part of the overall burden. This interpretation aligns with contemporary injury etiology frameworks, which conceive injury as the result of a dynamic, recursive, and cumulative process over time, where repeated exposures, adaptations, and injury history modify future risk [26]. In this sense, injury recurrence signals divergent competitive trajectories, ranging from profiles with high availability to players with chaining of episodes and progressive loss of availability [27].

### 4.2. Preseason Adiposity as an Integrated and Physiologically Plausible Phenotype

A strength of the study is the high internal coherence of preseason anthropometry. Body fat percentage was strongly associated with central (subscapular and abdominal) and peripheral skinfolds, reinforcing that these measurements capture a stable construct of adiposity and distribution. Complementarily, PCA supports this interpretation by condensing a large part of body variability into a dominant axis compatible with a robust adiposity gradient. This finding fits with the literature describing differentiated body profiles in professional basketball according to role and game demands, with greater mass and relative adiposity in interior players and lighter profiles in perimeter players, which may translate into differences in mechanical efficiency and energetic cost [6,7,8,9]. Likewise, a relationship between adiposity, testosterone, and performance has been described in professional players, supporting that body composition is related to relevant functional variables [28]. Although indices such as FFMI have been proposed as a contextual reference for fat-free mass in athletes [10], in this work, the focus on adiposity is coherent with the observed outcome and with the structural phenotype that emerges from the data.

### 4.3. Influence of Injury Outcome Definition on Detection of Associations

In bivariate analyses, preseason adiposity did not show a clear association with observed days lost or with the total number of injuries, but it did emerge more consistently when redefining the outcome as high injury burden (≥3 injuries). This apparent discrepancy can be interpreted in a physiologically and methodologically coherent way: adiposity would not necessarily increase the probability of an isolated event linearly but rather increase the probability of entering the recurrence subgroup, that is, players who “chain” episodes during the season. This reading is consistent with current models that understand risk as the result of interactions between predisposition, exposure, and accumulated consequences, including the role of previous injuries and their sequelae on load tolerance [26,27]. In other words, the ≥3 injury threshold acts as a detector of the most clinically relevant pattern in terms of collective performance.

Additionally, there is methodological precedence in other sports for integrating body measurements into the identification of vulnerable subgroups. In soccer, relationships between body composition and injury have been described, as well as multivariate predictive approaches incorporating anthropometry within risk models [14,29]. In high-performance youth populations, anthropometric variables have also been observed to relate to higher injury probability [30]. Although the professional context presents particularities (loads, competitive congestion, and physical profile), these precedents support the plausibility that precompetitive body composition may be integrated into a risk stratification framework.

### 4.4. Clinical Interpretation of the Association Between Adiposity and High Injury Burden

Team-stratified logistic regression models showed a positive association between preseason body fat percentage and high burden (≥3 injuries), with higher effect estimates in players belonging to the first men’s team. A prudent and useful interpretation is twofold. On one hand, the direction of the effect is coherent with the physiological hypothesis: greater adiposity, especially central, could be associated with lower mechanical efficiency, greater relative joint load, and less margin to tolerate competitive congestion, calendar intensification, and load peaks [1,2,22,31]. On the other hand, the wide confidence intervals are compatible with the small number of cases, suggesting uncertainty regarding the exact magnitude of the effect. In small samples with infrequent events, conventional logistic regression may produce unstable estimates, and penalized alternatives (e.g., Firth) have been proposed for rare-event scenarios [32]. This does not invalidate the observed signal, but places its interpretation in terms of clinical relevance and need for replication in additional seasons or cohorts.

### 4.5. Plausible Mechanisms: Why Adiposity Could Be Associated with Injury Recurrence

Our results suggest that preseason adiposity may behave as a marker of vulnerability to injury accumulation, and several plausible, non-exclusive pathways may hypothetically explain this association. First, there is a mechanical pathway: greater adipose tissue is associated with higher total mass and changes in body distribution, which may increase the energetic cost and load on musculotendinous and joint structures during repeated high-demand actions (accelerations, decelerations, jumps, and changes in direction), especially under fatigue. In a sport with high competitive density and elevated neuromuscular demand such as basketball [3,5], small differences in cost per action, repeated over time, could increase the probability of predominantly muscular and ligamentous injuries, with special involvement of the lower extremity. Second, adiposity may act as an indirect indicator of lower functional reserve or “relative fitness” for the same total mass or role, fitting with load models where high exposure can be protective if sufficient capacity exists, but harmful if rapid peaks accumulate or if adaptive capacity is lower [29,30]. Finally, there is a metabolic and recovery pathway: adiposity, especially central, has been associated with hormonal and performance profiles in professional basketball [28], and small disadvantages in recovery (fatigue, sleep, inflammation, or endocrine markers) may amplify individual differences during congested periods, facilitating recurrence. However, nutritional status, energy availability, and metabolic markers were not directly measured in the present study, and therefore, these interpretations should be considered contextual hypotheses derived from existing literature.

Importantly, these mechanisms were not directly measured in the present study and are proposed only as biologically plausible hypotheses derived from the existing literature. These pathways do not claim causality, given the retrospective design, but they do provide biological coherence to the observed pattern, supporting that precompetitive adiposity could function as a marker of “trajectory risk” rather than of a single isolated event.

### 4.6. Body Composition, Energy Availability, and Tissue Recovery

From an applied sports nutrition perspective, the observed relationship between higher preseason adiposity and probability of high injury burden can also be interpreted as an indirect marker of imbalances in the energy availability-recovery dyad. In sports with high neuromuscular demand and competitive congestion, availability to train and compete is strongly conditioned by the ability to sustain effective recovery processes, in which total energy intake, macronutrient distribution, and overall diet quality may play a modulatory role.

In this context, a phenotype with higher adiposity at the beginning of the season could reflect, in some cases, suboptimal nutritional periodization during the preparatory period, including insufficient carbohydrate management relative to training load. This is particularly relevant in an intermittent sport such as basketball, where repeated high-intensity efforts require consistent replenishment strategies. Although this study did not quantify dietary intake, precompetitive body composition may serve as an accessible indicator of functional nutritional status before the start of the competitive calendar, capable of conditioning load tolerance and recurrence of injury episodes during the season.

Complementarily, it should be considered that injury risk in professional sport is not exclusively associated with excess adiposity. Contemporary literature has described the impact of low energy availability (LEA) and relative energy deficiency in sport (RED-S) on health, performance, and biological resilience parameters, with possible repercussions on recovery and, in certain contexts, risk of bone stress injury [33]. Likewise, nutrition may influence tissue repair processes: strategies aimed at favoring collagen synthesis, such as ingestion of gelatin enriched with vitamin C prior to mechanical stimulus, have been shown to increase markers associated with collagen synthesis [34]. Although these findings do not allow inference of direct prevention in our cohort, they provide plausibility to the idea that preseason constitutes a strategic window to optimize body profile and musculotendinous resilience before accumulated competitive stress. Finally, supplement use should be considered within an individualized global strategy, with quality control and evidence-based criteria, as reflected in the International Olympic Committee consensus [35].

### 4.7. Clinical Phenotypes

The clustering analysis identified two differentiated phenotypes: one with higher preseason adiposity (global and central) and higher injury burden, and another with more favorable profiles and lower burden. This finding is relevant because it emerges from an unsupervised method, without imposing thresholds or linear relationships, which reinforces its internal coherence and physiological plausibility. In addition, it is consistent with the proposal to move from the identification of isolated factors to the description of patterns and regularities derived from complex systems [27]. From an applied perspective, these results suggest that it may be more useful to identify integrated vulnerability profiles than to focus on a single variable, since the phenotype can integrate body composition, exposure, and history, facilitating individualized monitoring.

### 4.8. Practical Implications

The results suggest that an accessible and standardizable preseason marker (ISAK anthropometry) may be useful to stratify vulnerability to high injury burden, especially when the operational objective is to reduce recurrence (≥3 injuries) that compromises availability. The key is that the findings of this work are supported by multiple layers: internal coherence of the phenotype, latent structure, relationship with the most clinically relevant outcome, and replication of the pattern through an unsupervised method.

In practical terms, these results reinforce that precompetitive anthropometric monitoring should be integrated with individualized nutritional planning, prioritizing adequate energy availability and consistent recovery strategies to favor load tolerance and minimize repeated injury accumulation throughout the season.

### 4.9. Strengths and Limitations of the Study

Among the main strengths of the study is its conduct in a real professional high-performance environment, based on field research data obtained within the usual clinical and sporting context of a professional club. The use of official club medical records, together with standardized anthropometric assessment (ISAK) performed in preseason, confers high ecological validity and direct applicability to professional practice. Likewise, the definition of outcomes aligned with competitive impact (observed days lost and high injury burden) reinforces the operational relevance of the results. Finally, the combination of correlation analysis, PCA, and clustering is coherent with the expected collinearity among skinfolds and with a phenotypic approach aimed at identifying risk profiles in a real high-performance sport context.

The main limitations derive from design and sample size. First, the retrospective nature limits causal inferences and leaves open the possibility of residual confounding. Important contextual factors commonly associated with injury risk in professional basketball (such as playing position, competitive exposure (e.g., minutes played), accumulated training load, and previous injury history) were not systematically incorporated into the analytical models and may therefore have influenced the observed associations.

Second, observed days lost were calculated only for injuries with documented clearance, which may underestimate the total temporal burden. Finally, the ≥3 injury outcome generates a small number of cases, with wide intervals and potential instability of logistic estimation, especially in stratified analyses, so the magnitude of the OR should be interpreted with caution, maintaining focus on direction of effect and coherence of the pattern.

## 5. Conclusions

This study suggests that body composition assessed in preseason may be associated with the real impact of injury during the season in male professional basketball. Beyond the simple count of episodes, the results indicate that injury may acquire competitive relevance when it translates into loss of availability, with this impact concentrated in a small subgroup of players who accumulate repeated injuries and longer periods of absence.

From a functional perspective, these findings highlight the importance of interpreting injury not only as an isolated event, but as a process with cumulative consequences on player availability and collective team performance. In this context, outcomes based on days lost and injury recurrence may provide more informative indicators for decision-making than isolated incidents.

The high internal coherence among anthropometric adiposity indicators supports the idea that preseason body composition behaves as an integrated pattern of global and central adiposity. The observed association between greater precompetitive adiposity and the probability of presenting high injury recurrence suggests that this body composition profile may function as a potential marker of unfavorable injury trajectories, characterized by episode accumulation and lower sport availability throughout the season.

Overall, these results should be interpreted as preliminary observations derived from an exploratory cohort. Further studies with larger samples, multiple competitive seasons, and more comprehensive monitoring of exposure and contextual variables are needed to confirm these patterns. Nevertheless, standardized preseason anthropometry conducted in real high-performance environments may provide useful information for the early identification of players who could require closer monitoring and individualized preventive strategies aimed at preserving competitive availability.

## Figures and Tables

**Table 1:** Baseline descriptive statistics of preseason body composition.

Preseason Variable	Total Mean (SD)	Total Median [IQR]	First Team Mean (SD)	First Team Median [IQR]	Second Team Mean (SD)	Second Team Median [IQR]	*p*-Value
Body fat percentage (%)	11.2 (1.3)	11.1 [10.3, 12.1]	10.4 (2.1)	10.9 [9.7, 11.8]	12.5 (1.2)	12.6 [11.9, 13.3]	0.005
Sum of skinfolds (mm)	72.0 (15.4)	70.0 [60.0, 83.0]	65.1 (13.0)	65.5 [55.9, 70.9]	80.7 (11.8)	77.0 [72.9, 85.9]	0.004
Subscapular skinfold (mm)	8.8 (2.4)	8.4 [7.2, 10.0]	9.4 (1.8)	9.7 [7.6, 10.6]	10.4 (1.6)	10.0 [9.1, 11.4]	0.110
Abdominal skinfold (mm)	10.8 (3.3)	10.1 [8.5, 12.7]	10.8 (3.4)	10.6 [8.4, 13.4]	14.8 (4.0)	14.2 [13.2, 16.7]	0.020
Calf skinfold (mm)	6.4 (2.6)	6.0 [5.0, 6.8]	5.8 (1.2)	5.6 [5.0, 6.6]	7.4 (3.7)	6.5 [6.0, 7.8]	0.090

Values expressed as mean (SD) and median [IQR].

**Table 2:** Descriptive statistics of in-season injury burden.

Variable	Total	First Team	Second Team
Players with ≥1 time-loss injury, n	13	7	6
Total number of time-loss injuries, n	16	10	6
Total days lost, n	2221	1020	1201
Days lost per injury, median [IQR]	10.5 [5.8, 40.0]	17.5 [6.0, 37.0]	10.0 [6.5, 35.3]

Values expressed as n and median [IQR].

**Table 3:** Correlations between preseason body composition and in-season injury burden.

Variables	Observed Days Lost in Season	Number of Injuries in Season	Body Fat Percentage (Preseason)	Subscapular Skinfold (Preseason)	Abdominal Skinfold (Preseason)	Calf Skinfold (Preseason)
Observed days lost in season	-	0.34 (*p* = 0.147)	0.06 (*p* = 0.810)	0.07 (*p* = 0.777)	0.04 (*p* = 0.867)	0.00 (*p* = 0.992)
Number of injuries in season	0.34 (*p* = 0.147)	-	0.35 (*p* = 0.119)	0.35 (*p* = 0.122)	0.33 (*p* = 0.153)	0.27 (*p* = 0.246)
Body fat percentage (preseason)	0.06 (*p* = 0.810)	0.35 (*p* = 0.119)	-	**0.86 (*****p*** **< 0.001)**	**0.89 (*****p*** **< 0.001)**	**0.73 (*****p*** **< 0.001)**
Subscapular skinfold (preseason)	0.07 (*p* = 0.777)	0.35 (*p* = 0.122)	**0.86 (*****p*** **< 0.001)**	-	**0.85 (*****p*** **< 0.001)**	**0.66 (*****p*** **< 0.001)**
Abdominal skinfold (preseason)	0.04 (*p* = 0.867)	0.33 (*p* = 0.153)	**0.89 (*****p*** **< 0.001)**	**0.85 (*****p*** **< 0.001)**	-	**0.62 (*****p*** **= 0.001)**
Calf skinfold (preseason)	0.00 (*p* = 0.992)	0.27 (*p* = 0.246)	**0.73 (*****p*** **< 0.001)**	**0.66 (*****p*** **< 0.001)**	**0.62 (*****p*** **= 0.001)**	-

Pearson correlations r (*p*-value). Values in bold indicate statistically significant correlations.

**Table 4:** Team-stratified logistic regression: high injury burden (≥3 injuries in season) according to preseason body fat percentage.

Team	Players (n)	Cases (≥3 Injuries), n	Odds Ratio (OR)	95% CI	*p*
First team	15	3	6.83	1.60–52.62	0.038
Second team	13	2	2.79	1.12–26.41	0.041

Odds Ratio (OR) per +1 percentage point increase in preseason body fat percentage.

**Table 5:** Identified phenotypes: preseason body composition and in-season injury burden (means).

Phenotype	Body Fat Percentage (Preseason)	Subscapular Skinfold (Preseason, mm)	Abdominal Skinfold (Preseason, mm)	Number of Injuries in Season	Observed Days Lost in Season
Higher injury burden phenotype	11.8	9.8	12.0	1.6	17.2
Lower injury burden phenotype	10.5	7.6	8.7	0.6	3.9

## Data Availability

The data presented in this study are available upon request from the corresponding author.

## Abbreviations

The following abbreviations are used in this manuscript:

ACB	Asociación de Clubs de Baloncesto (Association of Basketball Clubs, Spanish professional basketball league)
BIVA	Bioelectrical Impedance Vector Analysis
BMI	Body Mass Index
CI	Confidence Interval
FFMI	Fat-Free Mass Index
IOC	International Olympic Committee
ISAK	International Society for the Advancement of Kinanthropometry
IQR	Interquartile Range
LEA	Low Energy Availability
LEB Plata	Liga Española de Baloncesto Plata (Spanish Third-Tier Professional Basketball League)
OR	Odds Ratio
PCA	Principal Component Analysis
RED-S	Relative Energy Deficiency in Sport
